# A nomogram model for predicting seizure control in status epilepticus: development and risk factor analysis

**DOI:** 10.3389/fneur.2026.1849084

**Published:** 2026-08-11

**Authors:** Zhenyu Huang, Shenggen Chen, Hanbin Lin, Yuying Zhang, Luyan Wu, Wanhui Lin, Chaofeng Zhu, Huapin Huang

**Affiliations:** 1 Department of Neurology, Fujian Medical University Union Hospital, Fuzhou, China; 2 Fujian Key Laboratory of Molecular Neurology, Fuzhou, China; 3 Department of Geriatrics, Fujian Medical University Union Hospital, Fuzhou, China

**Keywords:** nomogram, prognosis, risk factor, seizure control, status epilepticus

## Abstract

**Objectives:**

To explore the risk factors impacting control of seizures in status epilepticus (SE) and to construct a valid nomogram for predicting the prognosis.

**Methods:**

In this retrospective study, we analyzed the patients with SE who were hospitalized in Fujian Medical University Union Hospital from January 2017 to December 2022. Two separate groups of data are created: one for model development and the other for model validation.

**Results:**

A total of 272 patients with SE were enrolled in this study, of which 89 had poorly-controlled seizures at discharge. Six potential risk predictors were identified through LASSO regression. After validation by univariate logistic regression (*p* < 0.01) and multivariate logistic regression (*p* < 0.05), four of the predictors: the classification of SE, the seizure type, respiratory complications, and circulatory complications, were confirmed as independent risk factors affecting seizure control in SE patients. The AUC of the predictive model was 0.829, that of validation was 0.710.

**Conclusion:**

The proposed nomogram, constructed based on four independent risk factors, exhibits acceptable discriminative ability and clinical benefit. The risk of poorly-controlled seizures in individual SE patients can be predicted through the nomogram. The model may facilitate the early and timely identification of high-risk patients.

## Highlights

- Developed a nomogram to predict individual SE patients’ poor seizure control risk.
- Nomogram showed clinical benefit via DCA and acceptable calibration in both development/validation cohorts.
- The correlation between age and epileptic seizures was insufficient to develop a nomogram for predicting short-term prognosis.
- Though duration of seizures was a significant risk factor, it was not suitable for building prognostic prediction models.

## Introduction

Status epilepticus (SE) is a severe neurological emergency with an annual incidence ranging from 9.9 to 41 per 100,000 people (1–5). It has characteristics of significant morbidity and mortality in the short term (6). The overall mortality associated with SE approaches 20%, with generalized convulsive status epilepticus representing about 45–74% of all cases (7, 8). The case fatality rate ranges from 5 to 46% (6, 9). Prolonged seizures may cause neuronal damage, permanent destruction to vulnerable regions in the hippocampus, thalamus, and neocortex, and in some cases lead to brain tissue hypoxia, elevated intracranial pressure, and cerebral herniation (10, 11). Moreover, the economic and psychological strain imposed on families and society due to poorly-controlled SE presents a significant concern. Therefore, the primary treatment for SE is seizure control, and seizures must be controlled as reasonably as possible. Long-term intervention strategies must be implemented for patients deemed challenging to control seizures, and anti-epileptic drugs should not be haphazardly terminated or arbitrarily reduced (12). Early prediction of individual patients’ prognosis in SE is a crucial step that can effectively inform treatment strategies. Previous research has indicated that seizure type and the duration of seizures are recognized risk factors affecting the prognosis of SE (13). Age, impairment of organ function, and history of epilepsy are also related to controlling seizures. We can detect cases that been improperly treated by assessing the risks that affect seizure control. There have been many tools to predict the outcome of SE patients, such as scale scores, electroencephalograms (EEG), imaging results (14–16). However, these predictive tools are constrained from universal implementation across all regions by either subjective biases of technical personnel or limitations of specialized equipment. Although biomarkers such as neuron specific enolase, the protein S-100B, and glial fibrillary acidic protein in serum are all considered to be correlated with SE (17–20). They similar encounter challenges posed by the technical requirements of detection methodologies.

The nomogram has been a popular prediction tool in recent years. It assigns scores to multiple variables, enabling the prediction of the probability of individual outcome events based on numerical values, demonstrating its superiority in visualizing personalized calculations for predicting the prognosis of individual patients, thereby exhibiting substantial clinical value in practical medical applications. This study screened the common serum biochemical indices along with clinical data, sought to identify risk factors affecting SE development, and created a nomogram model to predict the prognosis of individual SE patients.

## Methods

### Study participants

This study retrospectively collected the clinical data of patients with SE hospitalized in Fujian Medical University Union Hospital from January 2017 to December 2022. The inclusion criteria included: (1) all patients diagnosed with epileptic status at discharge; (2) the diagnosis of SE referred to the diagnostic criteria which the International League Against Epilepsy (ILAE) reported in 2015 (21); (3) the medical record was complete and without omissions. Exclusion criteria included: (1) there was unclear description of medical records and was inability to obtain relevant medical history evidence by follow-up; (2) patients or family members who had doubts about this study and refused to participate. The study was approved by the Ethics Committee of the Fujian Medical University Union Hospital. The study was conducted in accordance with the Declaration of Helsinki, and all participants provided informed consent for the use of their medical records.

### Data collection

The clinical data were collected from the data platform of Fujian Medical University Union Hospital, including demographics, the course of treatment, and the results of laboratory tests. While this allowed standardized data collection, the results might be influenced by regional healthcare disparities and patient selection bias. The results of CT scans and EEG were sourced from the relevant department. The seizure type and classification of epilepsy were determined according to the same criteria as previously mentioned. Seizure duration was defined as the time from the onset of SE to recovery of consciousness level to baseline. If the patient experienced more than one seizure while in hospital, the duration of the longest one seizure was recorded for the analysis. Based on clinical evidence, we classified the etiology of SE into six categories: structural, inflammatory, infectious, metabolic, genetic, and cryptogenic etiologies. And all laboratory tests collected in this study were performed on admission or for the first time after the onset of SE. Serious diseases or malignant tumors in many physiological systems after the occurrence of SE were recorded as different system complications. The classification and ICD codes of these complications are provided in Extended Data Table 1, and the severity criteria for system complications are detailed in Extended Data Table 2. Respiratory complications included respiratory failure, severe pneumonia, pneumothorax, etc. Cardiovascular complications included circulatory failure, heart failure, moderate or severe myocardial enzyme abnormalities, and severe arrhythmia. Digestive complications included moderate or severe liver function damage, gastrointestinal bleeding, etc. Urinary complications included moderate or severe renal function damage and urinary tract infection. Endocrine complications included three or more electrolyte disturbances, moderate and more irregular serum sodium or potassium, pituitary hormone abnormalities, etc. We excluded cases with missing information from relevant analyses.

**Table 1:** Patients characteristics of well-controlled vs. poorly-controlled group.

Characteristics	Development datasets (*n* = 180)		Invalidation datasets (*n* = 92)	
	Well-controlled (*n* = 121)	Poorly-controlled (*n* = 59)	Well-controlled (*n* = 62)	Poorly-controlled (*n* = 30)
Demographics				
Gender (male)	73 (60.3%)	30 (50.8%)	44 (71.0%)	12 (40.0%)
Age (yr)	1.0–86.0	2.9–98.0	0.2–86.0	0.3–88.0
Age of onset (yr)	0.2–86.0	2.9–98.0	0.2–86.0	0.2–88.0
History of epilepsy	41 (33.9%)	11 (18.6%)	25 (40.3%)	11 (36.7%)
Seizure type				
CSE	109 (90.1%)	32 (54.2%)	52 (83.9%)	21 (70.0%)
NCSE	12 (9.9%)	25 (42.4%)	7 (11.3%)	6 (20.0%)
Mix	0	2 (3.4%)	3 (4.8%)	3 (10.0%)
Classification				
SE	90 (74.4%)	31 (52.5%)	49 (79.0%)	14 (46.7%)
RSE	28 (23.1%)	13 (22.0%)	10 (16.1%)	7 (23.3%)
SRSE	3 (2.5%)	15 (25.4%)	3 (4.8%)	9 (30.0%)
Seizure duration (h)	0.8–408	0.17–496	0.08–386	0.17–576
Loss of consciousness	71 (58.7%)	48 (81.4%)	37 (59.7%)	23 (76.7%)
Fever	49 (40.5%)	32 (54.2%)	16 (25.8%)	8 (26.7%)
Abnormal results of laboratory				
White blood cell count	29 (24.0%)	21 (35.6%)	21 (33.9%)	19 (63.3%)
Lymphocyte count	25 (20.7%)	18 (30.5%)	16 (25.8%)	12 (40.0%)
Lymphocyte percentage	81 (66.9%)	43 (72.9%)	45 (72.6%)	23 (76.7%)
Neutrophil count	51 (42.1%)	29 (49.2%)	32 (51.6%)	17 (56.7%)
Neutrophil percentage	58 (47.9%)	36 (61.0%)	41 (66.1%)	23 (76.7%)
Albumin	23 (19.0%)	15 (25.4%)	8 (12.9%)	10 (33.3%)
Globulin	18 (14.9%)	11 (18.6%)	11 (17.7%)	5 (16.7%)
A/G	26 (21.5%)	15 (25.4%)	8 (12.9%)	5 (16.7%)
ALT	22 (18.2%)	14 (23.7%)	9 (14.5%)	5 (16.7%)
AST	49 (40.5%)	32 (54.2%)	23 (37.1%)	7 (23.3%)
ALP	33 (27.3%)	16 (27.1%)	20 (32.3%)	10 (33.3%)
GGT	36 (29.8%)	24 (40.7%)	24 (38.7%)	8 (26.7%)
Urea	46 (38.0%)	22 (37.3%)	20 (32.3%)	14 (46.7%)
Creatinine	22 (18.2%)	14 (23.7%)	11 (17.7%)	9 (30.0%)
Potassium	22 (18.2%)	7 (11.9%)	12 (19.4%)	6 (20.0%)
Sodium	28 (23.1%)	14 (23.7%)	7 (11.3%)	8 (26.7%)
Urine ketone bodies	27 (22.3%)	4 (6.8%)	16 (25.8%)	7 (23.3%)
Positive CT	39 (32.2%)	13 (22.0%)	27 (43.5%)	10 (33.3%)
EEG				
Normal EEG	18 (14.9%)	4 (6.8%)	10 (16.1%)	5 (16.7%)
Abnormal background without epileptiform discharges	52 (43.0%)	26 (44.1%)	38 (61.3%)	8 (26.7%)
Epileptiform discharges	47 (38.8%)	29 (49.2%)	14 (22.6%)	17 (56.7%)
Mechanical ventilation	37 (30.6%)	30 (50.8%)	13 (21.0%)	15 (50.0%)
Etiology				
Structural etiology	47 (38.8%)	34 (57.6%)	27 (43.5%)	13 (43.3%)
Genetic etiology	5 (4.1%)	0	1 (1.6%)	0
Infectious etiology	33 (27.3%)	15 (25.4%)	11 (17.7%)	7 (23.3%)
Metabolic etiology	4 (3.3%)	2 (3.4%)	3 (4.8%)	1 (3.3%)
Inflammatory etiology	10 (8.3%)	6 (10.2%)	2 (3.2%)	2 (6.7%)
Cryptogenic etiology	22 (18.2%)	2 (3.4%)	18 (29.0%)	7 (23.3%)
Complications				
Respiratory system	57 (47.1%)	46 (78.0%)	34 (54.8%)	25 (83.3%)
Cardiovascular system	30 (24.8%)	34 (57.6%)	14 (22.6%)	12 (40.0%)
Digestive system	34 (28.1%)	23 (39.0%)	17 (27.4%)	10 (33.3%)
Urinary system	18 (14.9%)	20 (33.9%)	11 (17.7%)	5 (16.7%)
Hematological system	30 (24.8%)	17 (28.8%)	17 (27.4%)	14 (46.7%)
Endocrine system	36 (29.8%)	26 (44.1%)	18 (29.0%)	15 (50.0%)

Abbreviations: CSE, convulsive status epilepticus; NCSE, non-convulsive status epilepticus; Mix, mixed seizure; SE, status epilepticus; RSE, refractory status epilepticus; SRSE, super-refractory status epilepticus; A/G, albumin/globulin; ALT, alanine aminotransferase; AST, Aspartate aminotransferase; ALP, alkaline phosphatase; GGT, gamma glutamyl transpeptidase; EEG, electroencephalogram.

**Table 2:** Univariate logistic regression analysis of clinical candidate predictors.

Variables	Univariate analysis		*P*
	OR	95% CI	
Age of onset, years (continuous)	1.018	1.007 to 1.029	0.001
Seizure duration, hours (continuous)	1.002	1.001 to 1.002	2.17*10^−4^
Seizure type			2.09*10^−6^
CSE vs. Mix	0.198	0.046 to 0.854	
NCSE vs. Mix	0.979	0.210 to 4.571	
Classification of status epilepticus			1.45*10^−6^
SE vs. SRSE	0.081	0.031 to 0.210	
RSE vs. SRSE	0.132	0.046 to 0.374	
Cardiovascular complications (No vs. Yes)	0.296	0.173 to 0.506	8.63*10^−6^
Respiratory complications (No vs. Yes)	0.251	0.139 to 0.454	4.81*10^−6^

CSE, convulsive status epilepticus; NCSE, non-convulsive status epilepticus; Mix, mixed seizure; SE, status epilepticus; RSE: refractory status epilepticus; SRSE: super-refractory status epilepticus; OR, odds ratio; CI, confidence intervals.

### VEEG analysis

The EEG examination was recorded by video-EEG (VEEG), and the monitoring period was more than 2 h. The initial recording was chosen if there were several. The VEEG data were scrutinized and classified as “normal EEG,” “abnormal background without epileptiform discharges,” or “epileptiform discharges” based on the original VEEG recordings or reports.

### Brain CT analysis

In the emergency or intensive care setting, Computed tomography (CT) was performed as the first-line screening neuroimaging for patients to rule out structural abnormalities, whereas MRI was not universally available due to longer acquisition time, poor patient tolerance, and incompatibility with monitoring equipment. Therefore, this study only used CT findings for imaging analysis. The CT reports were reviewed and categorized as “Negative” or “Positive.” “Positive” indicated that the CT revealed structural abnormalities, including extensive brain edema, cerebral hemorrhage focus and cerebral infarction lesion consistent with epileptic discharge. “Negative” referred to CT findings without structural abnormalities.

### Statistical analysis

SPSS 25.0 software (IBM Corporation) and Stata version 16 were used for data analysis. The laboratory tests were converted into binary variables according to whether they were abnormal or not. For continuous variables, the range (minimum value - maximum value) was presented to reflect the full distribution of data across the study population. For categorical variables, counts and percentages [n (%)] were used to describe their constituent ratios. A two-sided *p* < 0.05 was deemed significant. LASSO regression analysis, univariate and multivariate logistic regression analysis were used to identify factors that had an influence on the prognosis of SE.

### Development and validation model

Data from January 2017 to December 2020 were utilized as development datasets for model construction, whilst data from January 2021 to December 2022 served as validation datasets for internal validation. We screened all parameters included in the study through LASSO regression analysis and obtained some candidate factors. And all candidate factors with *p* < 0.05 in the univariate logistic regression analysis were included in the initial multivariate logistic regression analysis. Then, after eliminating non-significant parameters, the results of multivariate logistic regression analysis were used to develop a nomogram. The final model was the model corresponding to the minimum Akaike information criterion (AIC). In the nomogram model, the regression coefficient of each predictor was used to determine the proportion of scores. Ten points were assigned to the predictor with the highest regression coefficient, and the other predictors were given corresponding points based on weight. Different clinical manifestations resulted in different scores, which could predict the prognosis of SE after summed. The higher the scores got, the greater the risk of poorly-controlled seizures occurring. Subsequently, the model was evaluated based on nomogram calibration. Areas under the receiver operating characteristic curves (AUC) were calculated for evaluating discrimination, and we drew calibration curves and visualized the Hosmer-Lemeshow goodness-of-fit test for evaluating calibration. A *p*-value >0.05 indicates good calibration. At last, we used decision curve analysis (DCA) to evaluate the effectiveness of the model.

## Results

**Figure 1:**
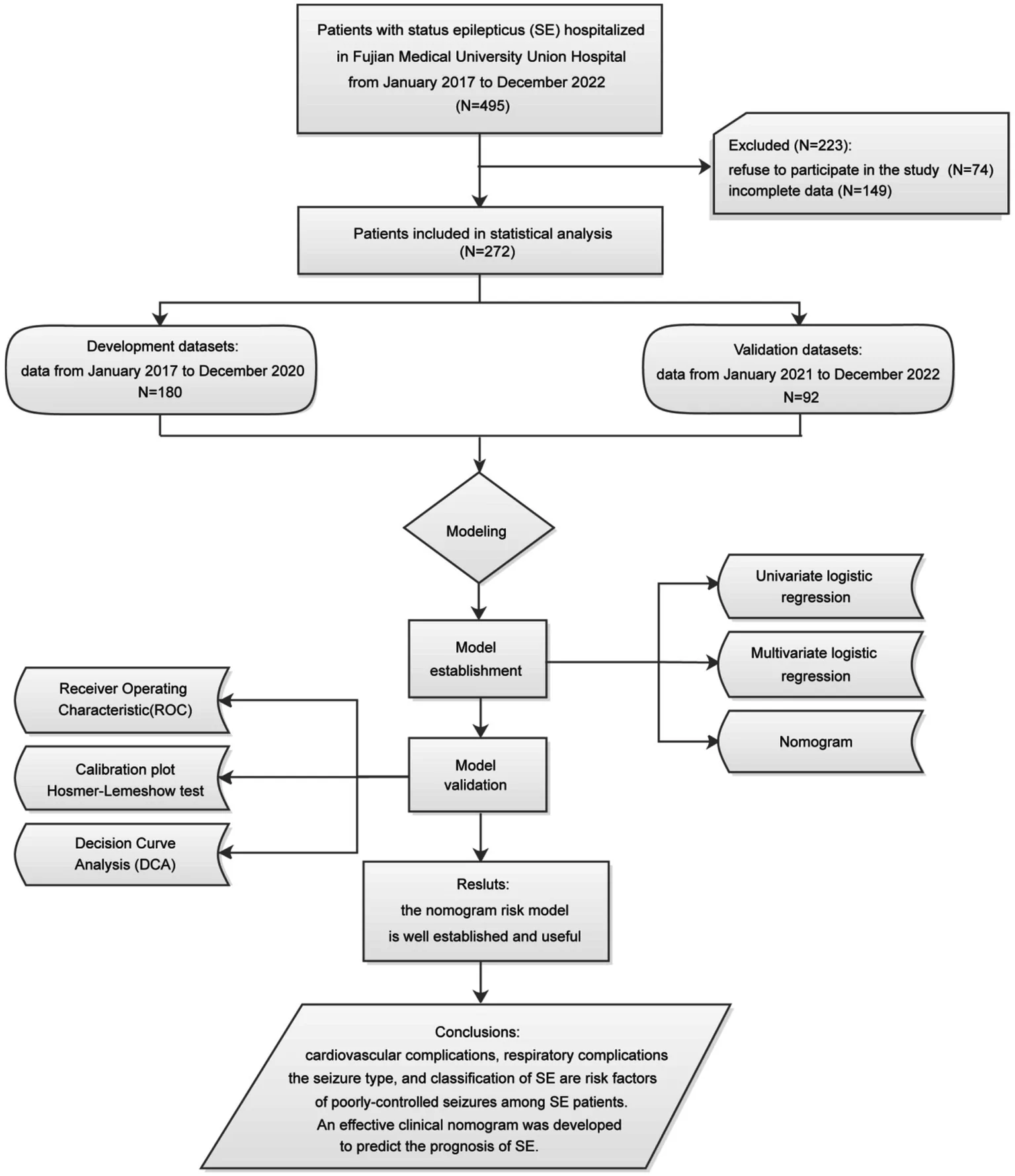
The flowchart of the study.

The clinical data of patients with SE hospitalized from January 2017 to December 2022 were reviewed, and a total of 495 patients who met the inclusion criteria were screened. According to the exclusion criteria mentioned above, 223 patients were excluded. The exclusion of data primarily occurred due to incomplete medical records. And 272 patients were finally included in this study for analysis. A total of 180 patients were included in the development datasets, and another 92 patients were included in the validation datasets for internal validation (Figure 1). According to whether they still had clinically observable epilepsy symptoms or not at discharge, patients were divided into well-controlled or poorly-controlled (Table 1). Table 1 shows the characteristics of the two groups within the development, validation, and entire datasets.

**Figure 2:**
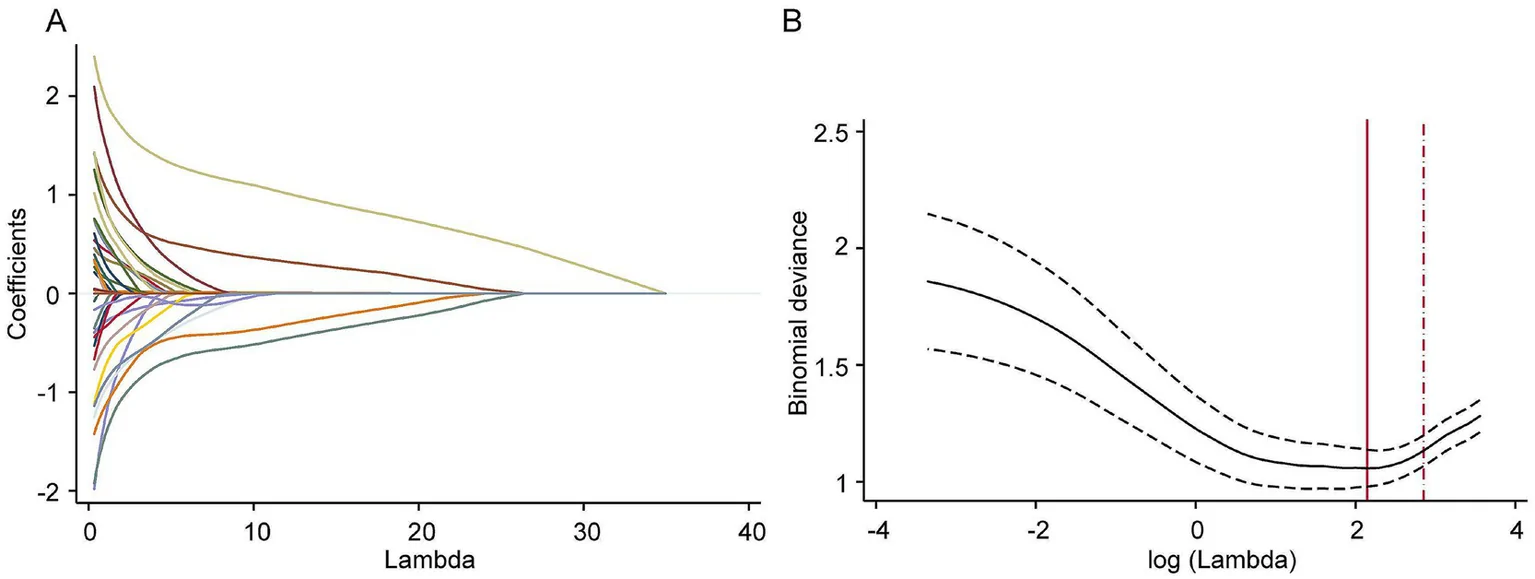
Predictor selection using the LASSO regression analysis with cross-validation. **(A)** A coefficient profile plot was created against the log (lambda) sequence. **(B)** Tuning parameter (lambda) selection of deviance in the LASSO regression. LASSO: Least absolute shrinkage and selection operator.

We used LASSO regression analysis on the 36 independent variables in development datasets for variable selection, with seizure control as the dependent variable (Figure 2).

And six potential risk factors were identified, including age of onset, seizure duration, seizure type, classification of SE, cardiovascular complications, and respiratory complications. Univariate logistic regression analysis was performed on these variables subsequently, and it showed that all six were *p* < 0.01 (Table 2).

Those six potential risk factors entered into the initial multivariable logistic regression for further analysis. After elimination, four of them were recorded in the final logistic regression model: seizure type, classification of SE, cardiovascular complications, respiratory complications (Table 3).

**Table 3:** Multivariate logistic regression analysis of clinical candidate predictors.

Variables	Univariate analysis		*P*
	OR	95% CI	
Seizure type			3.90*10^−5^
CSE vs. Mix	0.268	0.056 to 1.289	
NCSE vs. Mix	1.340	0.248 to 7.242	
Classification of status epilepticus			2.27*10^−4^
SE vs. SRSE	0.108	0.037 to 0.315	
RSE vs. SRSE	0.135	0.043 to 0.424	
Cardiovascular complications (No vs. Yes)	0.366	0.194 to 0.689	0.002
Respiratory complications (No vs. Yes)	0.419	0.205 to 0.855	0.017

CSE, convulsive status epilepticus; NCSE, non-convulsive status epilepticus; Mix, mixed seizure; SE, status epilepticus; RSE, refractory status epilepticus; SRSE, super-refractory status epilepticus; OR, odds ratio; CI, confidence intervals.

**Figure 3:**
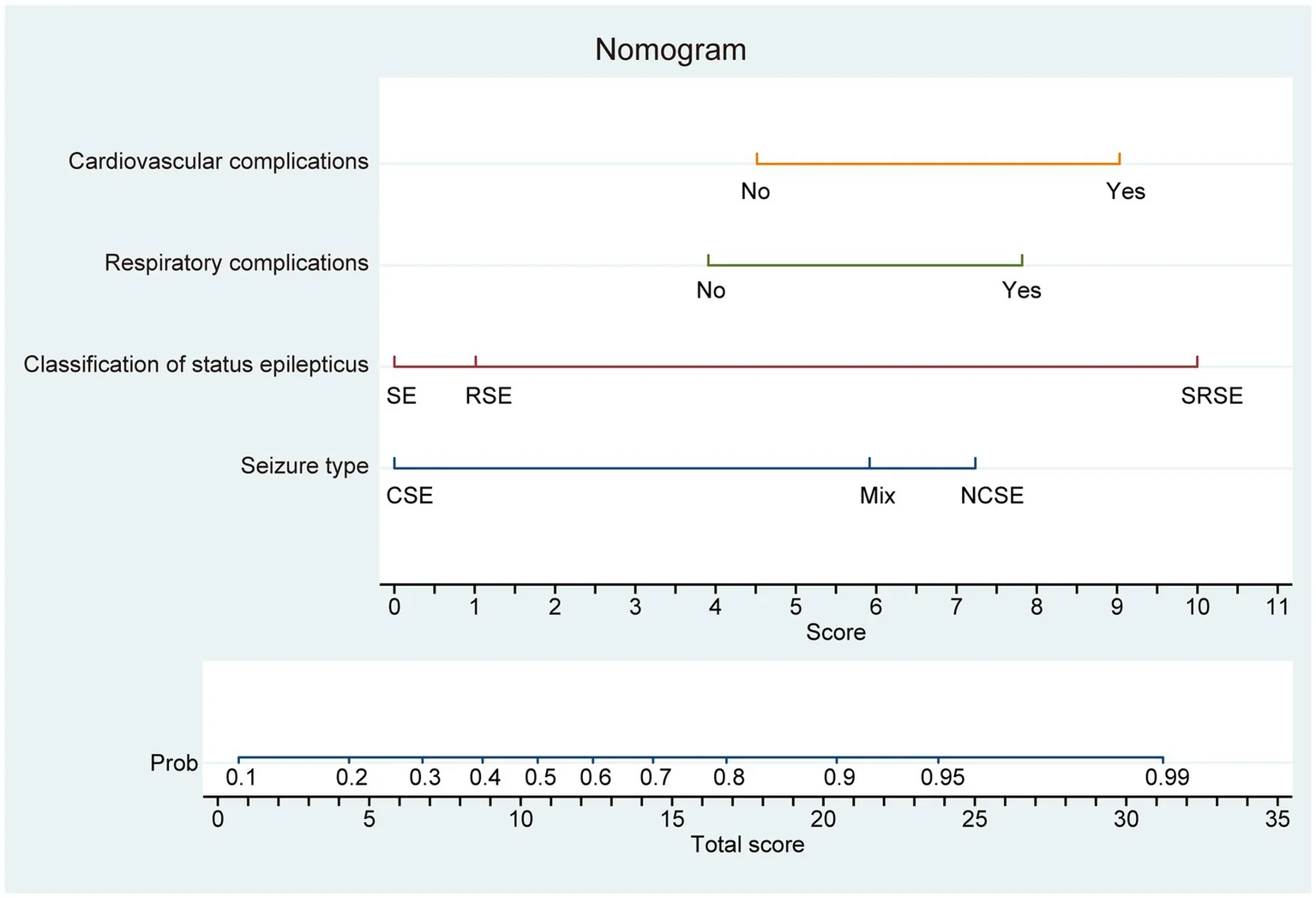
Nomogram for predicting poorly-controlled seizures in SE patients. CSE, convulsive status epilepticus; NCSE, non-convulsive status epilepticus; Mix: mixed seizure; SE, status epilepticus; RSE, refractory status epilepticus; SRSE, super-refractory status epilepticus.

They were all statistically significantly related to the prognosis of SE (*p* < 0.05). A model incorporating these six characteristics was created according to the multivariable logistic regression results, and a nomogram to predict the prognosis of SE was developed (Figure 3).

In the nomogram, the classification of SE was assigned 10 points. For each SE patient, we first identified the position of each variable on the corresponding axis, then we summed the points for each variable to form a total score. The total score axis was used to estimate the probability (ranging from 10 to 99%) of poorly-controlled SE for individual patients.

**Figure 4:**
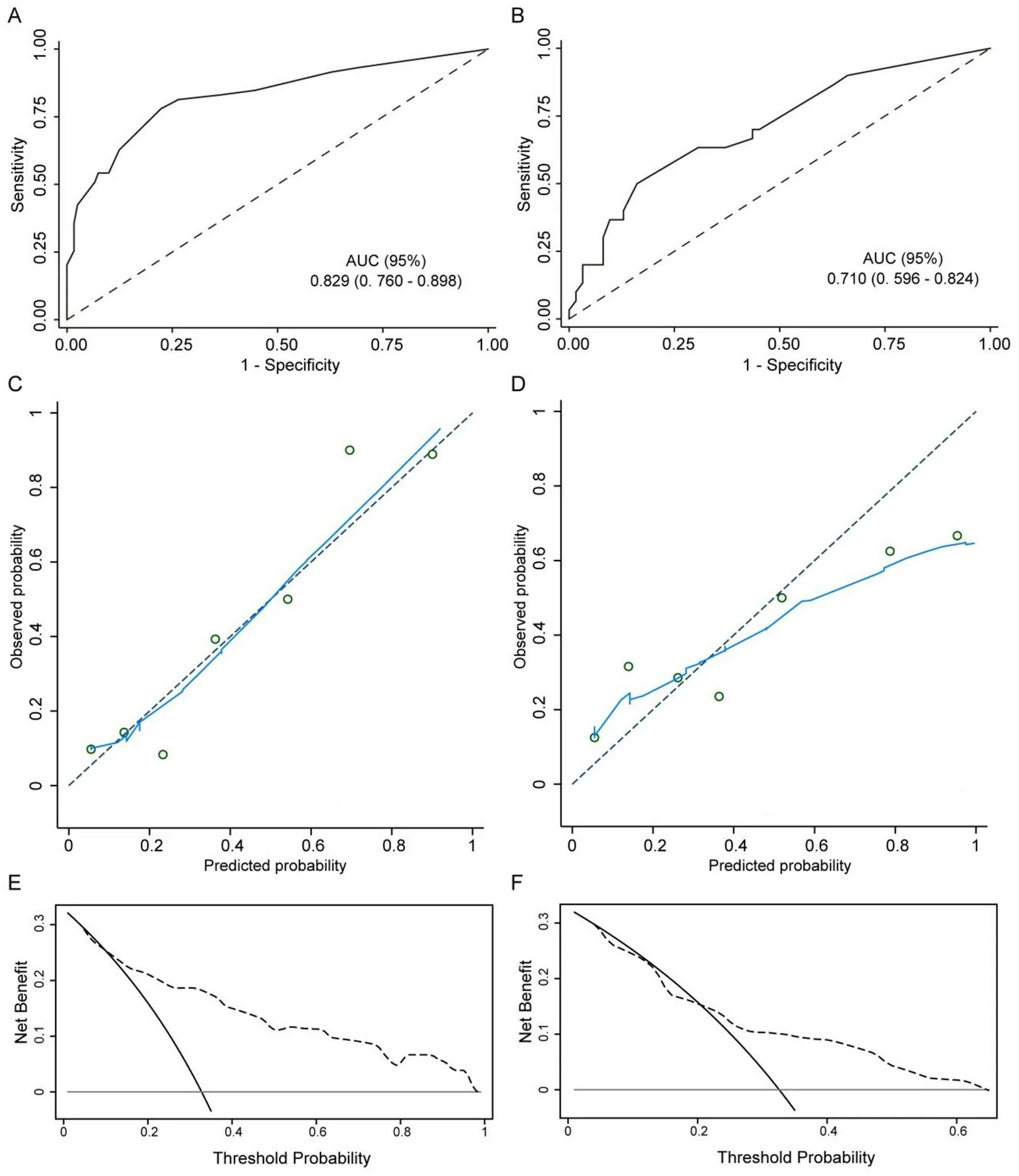
ROC curves of the nomogram in the development datasets **(A)**, and that in the validation datasets **(B)**. The AUC of the development datasets was 0.829 (95% CI: 0.760–0.898), and that of the validation datasets was 0.710 (95% CI: 0.596–0.824). The Hosmer-Lemeshow goodness-of-fit test was used to compare predicted probability and observed probability. Calibration curves in development datasets (*p* = 0.458) **(C)** and validation datasets (*p* < 0.001) **(D)**. DCA were calculated for evaluating the effectiveness of the nomogram model. Both the DCA of the development datasets **(E)** and the validation datasets **(F)** had clinical benefit values, showing acceptable clinical applicability. AUC: Area under the receiver operating characteristic curve; ROC: Receiver operating characteristic; DCA: Decision curve analysis.

The nomogram showed acceptable accuracy in predicting the risk of poorly-controlled patients with SE. The AUC of the development datasets was 0.829 (95%CI: 0.760–0.898), and the AUC of the validation datasets was 0.710 (95%CI: 0.596–0.824; Figures 4A,B). The calibration curve for the development cohort showed good agreement, while the calibration curve for the validation cohort showed some deviation at higher risk thresholds. The Hosmer-Lemeshow goodness-of-fit test χ^2^ statistics of the development datasets (*p* = 0.458) and the validation datasets (*p* < 0.001) showed the results of calibrations (Figures 4C,D). The suboptimal calibration in the validation cohort might be attributed to limited sample size (*n* = 92), which could reduce the test’s power to detect true calibration. Despite this, the DCA showed that the model provided net benefit across a wide range of threshold probabilities, indicating that the model had acceptable applicability within a certain range (Figures 4E,F).

## Discussion

This study reviewed 272 patients diagnosed with SE. The study found that the risk factors affecting the prognosis of SE included the seizure type, the classifications of seizures, respiratory complications, and cardiovascular complications. Over the years, there have been many studies on the risk factors and prognosis of SE. Most of the literature focused on the long-term outcomes, and the control of seizures within a single course has not been explored. Early control is believed to be a better guide for treatment than the long-term outcome (6, 22). There is a need for more aggressive treatment to be taken in clinic based on the possible short-term control (23). Using the set variables, a nomogram was developed in this study to predict the risk of uncontrolled seizures in individual SE patients.

SE was defined by the International League against Epilepsy in 2015 as a disorder brings on by either mechanisms causing excessively extended seizures or failure of the systems necessary for terminating seizures. Undertreatment of SE can lead to a progression of refractory status epilepticus (RSE) and super-refractory status epilepticus (SRSE). RSE is defined as SE that persists despite at least two appropriately dosed parenteral anti-epileptic drugs. Patients with RSE experience a longer hospital stay, a lesser return to baseline, and a higher death rate (24). While SRSE is defined as SE that either recurs upon weaning off of anesthetics or that lasts for at least 24 h following the start of continuous anesthetic treatment (10). The outcome is worse since there is more harm from uncontrollably occurring seizures. We counted the RSE and SRSE as one and 10 points in this study, respectively, which delimited their influence on individuals to a certain extent.

According to the criteria published in 2015 by ILAE, SE can be classified into convulsive status epilepticus (CSE) and non-convulsive status epilepticus (NCSE). Seizures originating from different brain lobes or functional areas may have different effects on individuals and therefore may have different long-term outcomes. We defined mixed seizures as patients who exhibited both convulsive and non-convulsive symptoms during a single course of hospitalization. This study indicated that seizure type played a significant role in predicting the progression of seizure control in SE patients. It took longer to control mixed seizures and NCSE rather than CSE, and patients with NCSE were found most difficult to be controlled well. Yuan Fang’s team summarized the 145 CSE patients and found that the mortality rate of NCSE after CSE was much higher than that of overall CSE, with a mortality of 18.9% (25). Since there are no motor symptoms, the EEG and other tests are mostly needed to support the diagnosis of NCSE (26). In certain instances, coma may conceal the existence of NCSE, leading to an incorrect or postponed diagnosis during the initial stages of the illness (27). These have a less effective therapeutic impact than individuals who received early treatment (28). The clinical manifestations of CSE are easier to distinguish, and can often be timely treated in the early stage of the disease. We believed this was one of the possible reasons that NCSE and mixed seizures take longer to control (29). In conclusion, type of SE is an important factor in evaluating the progression of seizure control in SE patients and is also an indispensable part in predicting the prognosis.

In this study, we classified SE into structural, inflammatory, immunological, metabolic, genetic, and cryptogenic etiologies. Structural damage such as encephalorrhagia, subdural hematoma might cause defect symptoms or irritant symptoms during the hospitalization. And the intracranial infection as an inflammatory etiology in the course of SE could cause more irritant symptoms. Other etiologies also had different manifestations. The different impacts on the brain lead to various degrees of influence on the occurrence and development of SE. Though the research evidence now available indicates a strong association between the etiology and long-term prognosis of SE, more study is required to investigate its relationship with seizure control. The impact of etiology on the short-term prognosis of SE is not sufficient to be applied to develop the nomogram model. Meanwhile, the CT examination results, as the main factor suggesting structural etiology, also revealed a weak correlation with seizure control.

Many studies have suggested that age affects the long-term outcome of SE. This study reached a similar conclusion by univariate analysis that age was an independent risk factor for seizure control in patients with SE. However, it also revealed that in the course of a single treatment, the correlation between seizure control and age was slightly weaker than seizure type and the classification of seizure, which was not enough to be applied to develop the nomogram model. Mendiratta’s team have found in their study that elderly SE patients have a higher mortality rate, mainly due to their higher risk of cerebrovascular accidents and central nervous system tumors, which themselves have high disability and mortality rates (30).

Older patients are more likely to experience recurrences of SE, disability, or possibly pass away from complications (31, 32). For the older patients, non-motor manifestations, including somnolence and clumsiness, can be more common than convulsive seizures (32). The possibility of other physiological system complications and adverse reactions to antiepileptic drugs is also higher for them than young people. These may explain the differences in age performance in univariate and multivariate logistic regression analyses. Similar results were shown in seizure duration. Patients with longer seizure duration have more severe irreversible brain damage, and their seizures are more difficult to control. It is indeed a significant risk factor, but it was not suitable for building models. We believe that it is because without the monitoring of EEG devices, it is usually impossible to determine the onset time and cessation time, so most patients find it difficult to determine the exact duration of SE, and there may be bias in statistical analysis. In the context of SRSE, we recognize that seizure duration is inherently reflected in the classification of SE. But the absence of a direct measure of seizure duration may still limit the model’s precision in SRSE patients, particularly when estimating individualized risk at extreme durations. Future prospective studies with standardized EEG monitoring and accurate onset time documentation are warranted to further refine the model for SRSE populations.

It is found that there are significant correlations among NSE, S100-B, cerebrospinal fluid tau protein, and some other serum biomarkers on the prognosis of SE patients (33–35). But several biomarkers are not widely used in clinical work due to backward equipment or technology. Consequently, we aimed to search for some more prevalent clinical laboratory indicators as candidate risk factors for SE. However, all of these parameters were excluded from the univariate and multivariate logistic regression analysis in the LASSO regression analysis. We held the view that the sensitivity or specificity of serum biochemical indices in evaluating the prognosis of individual patients was insufficient to be used in model development. Some of these serum biomarkers reflected the function of the digestive system and urinary system to a certain extent. So we concluded that their relationship to whether or not SE was well-controlled was minimal. We deemed that it is relevant to the drugs. Functional deficiencies in the liver or kidney are relative contraindications for some drugs. Such patients with these problems may have fewer available drugs for healing or may have delayed treatment (36).

Furthermore, our model suggested that circulatory and respiratory complications are significantly associated with predicting SE outcome. There were nine points for circulatory complications and eight points for respiratory complications. They promote each other in the pathophysiological evolution. The presence of comorbidities and SE complications are other factors that contribute to worse outcomes (37). Respiratory issues impair pulmonary ventilation, triggering hemodynamic instability and circulatory dysfunction. Conversely, severe arrhythmias or cardiac structural abnormalities induce myocardial ischemia, reducing pulmonary arterial blood flow and worsening respiratory compromise. This vicious cycle contributes to pulmonary encephalopathy or hypoxic–ischemic encephalopathy, disrupting neuronal metabolism and exacerbating seizures. Clinically, this highlighted the need to address both seizure control and underlying precipitating factors at disease onset.

We acknowledge that the AUC decreased from 0.829 in the development cohort to 0.710 in the validation cohort. This reduction is not unexpected, as some degree of performance attenuation during internal validation is inherent to predictive models, and the smaller validation sample size (*n* = 92) may have further reduced the stability of the estimate. Despite this, an AUC of 0.710 indicates acceptable discriminative ability. The Hosmer-Lemeshow test yielded *p* < 0.001, suggesting deviation from perfect calibration in the validation cohort. Visual inspection of the calibration plot showed that the predicted probabilities were generally consistent with observed frequencies across most of the risk range, although the model slightly overestimated risk at higher thresholds (approximately >0.6). However, this deviation should be interpreted cautiously, as the limited sample size may make the calibration curve in this region particularly sensitive to extreme cases. The DCA confirmed net clinical benefit across a meaningful range of threshold probabilities. Larger independent cohorts are needed for further external validation.

Our study had several strengths. First, risk factors for poor outcomes in SE patients were identified by our study, and the findings were visualized through the creation of a nomogram. The nomogram provides a more specific prediction for individual SE patients. Secondly, the nomogram was developed based on clinical data that are easy to determine. This prediction model can be easily used to clinical work and is appropriate for SE patients. Moreover, the model behaved well in the development and validation datasets to lend credibility to its utility. The use of predictive tools can help clinicians to identify patients with poor prognosis early in the progress of disease, to develop more personalized treatment plans for them.

Despite these strengths, several limitations of this study should be acknowledged. First of all, this study was subject to the limitation of a single-center, Chinese hospital-based cohort. Although our sample included 272 patients with SE, the results may not fully generalize to other ethnic groups or healthcare settings. Second, the retrospective design inherently limited our ability to establish causality, as unmeasured or unobserved confounding factors might influence the association between seizure control and the risk factors. We might miss some underlying factors in the univariate or multivariate logistic regression analysis. Third, parts of our patients were referred from other hospitals, and thus the accurate time of onset and the exact duration of SE could not be obtained. While some variable data were based on medical records, which might lead to information bias. Finally, the actual duration of NCSE in some patients might be longer than we found because their condition made them uncooperative in completing the EEG examination in the beginning. Further prospective studies based on large samples are needed to validate the nomogram and to further elucidate the risk factors affecting the prognosis of SE.

## Conclusion

This study found that seizure type, classification of SE, cardiovascular complications, and respiratory complications were the key risk factors of poorly-controlled seizures among SE patients. An effective clinical nomogram was developed to predict the prognosis of SE. The proposed nomogram achieved potential for clinical utility in the prediction of seizure control. The use of this model can help clinicians identify patients with poor prognosis early in the progress of disease and initiate early individualized treatment for them. Given the calibration concerns in the validation cohort, further external validation in larger cohorts is warranted.

## Data Availability

The raw data supporting the conclusions of this article will be made available by the authors, without undue reservation.
